# Associations Between Smartphone and Social Media Use and Mental Well-Being Among Adults in Saudi Arabia: A Cross-Sectional Survey

**DOI:** 10.7759/cureus.113180

**Published:** 2026-07-22

**Authors:** Majed N Alanizy, Saad E Alsubaie, Marwan S Salamah, Mohammed A Jan, Ayman A Alamri, Anas M Alothaim, Najlaa M Al Sudairy

**Affiliations:** 1 Family Medicine, Ministry of National Guard Health Affairs, Madinah, SAU; 2 Family Medicine, Al-Madinah Al-Munawarah Health Cluster, Madinah, SAU; 3 Family Medicine, Ministry of National Guard Health Affairs, Jeddah, SAU

**Keywords:** digital health, mental well-being, problematic smartphone use, saudi arabia, smartphone use, social media, who-5 well-being index

## Abstract

Background

Smartphone and social media use have become integral components of modern life; however, concerns have increased regarding their potential influence on psychological well-being. Although excessive digital media use has been associated with depression, anxiety, and sleep disturbances, evidence regarding its relationship with positive mental well-being remains limited, particularly in populations with high levels of smartphone and social media engagement. We aimed to evaluate the association between smartphone use, social media behaviors, and mental well-being among adults in Saudi Arabia.

Methods

We conducted a cross-sectional survey of adults aged 18 years or older residing in Saudi Arabia between January and March 2026. Participants completed an anonymous electronic questionnaire assessing demographic characteristics, smartphone and social media behaviors, lifestyle factors, and mental well-being. The primary outcome was mental well-being measured using the World Health Organization Five Well-Being Index (WHO-5), analyzed both as a continuous score and as poor mental well-being (WHO-5 score <50). Associations between digital media behaviors and mental well-being were assessed using analysis of variance, correlation analyses, and multivariable logistic regression models adjusted for demographic and lifestyle factors.

Results

A total of 1,248 participants were included in the analysis. The mean (±SD) age was 30.8±9.4 years, and 691 participants (55.4%) were women. The median daily smartphone use was 5.8 hours (interquartile range, 3.9-8.2), and 447 participants (35.8%) reported ≥5 hours of daily social media use. Mean WHO-5 scores declined progressively with increasing smartphone use, from 72.4±15.3 among participants using smartphones for <2 hours/day to 50.8±19.6 among those using smartphones for >8 hours/day (P<0.001). The prevalence of poor mental well-being increased from 13 of 106 participants (12.3%) in the <2 hours/day group to 125 of 254 participants (49.2%) in the >8 hours/day group (P<0.001).

After multivariable adjustment, smartphone use >6 hours/day was associated with higher odds of poor mental well-being (adjusted odds ratio (OR), 2.41; 95% confidence interval (CI), 1.77-3.28). Additional independent associations were observed for social media use ≥5 hours/day (adjusted OR, 2.05; 95% CI, 1.48-2.84), frequent social comparison on social media (adjusted OR, 2.36; 95% CI, 1.73-3.20), and smartphone use before bedtime (adjusted OR, 1.69; 95% CI, 1.18-2.42). Regular physical activity was associated with lower odds of poor mental well-being (adjusted OR, 0.71; 95% CI, 0.53-0.95). Problematic smartphone behavior demonstrated the strongest inverse correlation with WHO-5 scores (r=-0.56, P<0.001).

Conclusions

Among adults in Saudi Arabia, greater smartphone and social media engagement were associated with poorer mental well-being, with a graded relationship observed between increasing smartphone use and declining WHO-5 scores. Problematic smartphone behaviors, social comparison, and bedtime smartphone use emerged as important behavioral correlates of reduced well-being, whereas physical activity was associated with more favorable psychological outcomes. Longitudinal studies are needed to clarify causal relationships and determine whether interventions targeting digital media habits can improve mental well-being.

## Introduction

The widespread adoption of smartphones and social media has transformed the way individuals communicate, access information, work, and engage in daily activities [1,2]. Over the past decade, smartphone ownership has reached unprecedented levels worldwide, with digital technologies becoming deeply integrated into personal, educational, and professional life [2,3]. Social media platforms, including Instagram, Snapchat, X (formerly Twitter), TikTok, and WhatsApp, have further expanded opportunities for social interaction and information sharing [1-4]. While these technologies provide substantial social, educational, and economic benefits, growing concerns have emerged regarding their potential impact on mental health and psychological well-being [3,5].

Mental well-being is a fundamental component of overall health. It extends beyond the absence of mental illness to encompass positive emotional functioning, resilience, life satisfaction, and the ability to cope with daily stressors [3,6]. Increasing evidence suggests that excessive smartphone use and prolonged social media engagement may adversely affect mental well-being through multiple behavioral and psychological mechanisms [1,3,7]. Proposed pathways include disrupted sleep, reduced physical activity, increased sedentary behavior, compulsive smartphone checking, fear of missing out (FOMO), cyberbullying, information overload, and frequent exposure to idealized social comparisons. Together, these factors may contribute to increased psychological distress, reduced life satisfaction, and diminished overall well-being [3-8].

Previous observational studies have reported associations between excessive screen time and symptoms of depression, anxiety, stress, loneliness, and poor sleep quality. However, the existing literature remains heterogeneous, with considerable variation in study populations, definitions of problematic smartphone use, assessment tools, and outcome measures [9-16]. Furthermore, many studies have focused primarily on psychiatric symptoms rather than positive mental well-being, despite increasing recognition that well-being represents a distinct and equally important dimension of mental health. The use of validated instruments such as the World Health Organization Five Well-Being Index (WHO-5) enables assessment of positive psychological functioning. It provides a more comprehensive evaluation of mental health than symptom-based measures alone [17].

Saudi Arabia represents a particularly relevant setting in which to investigate these relationships. The country has one of the highest rates of smartphone ownership, internet accessibility, and social media engagement globally, with digital platforms playing a central role in communication, education, entertainment, and commerce. Rapid digital transformation, coupled with widespread reliance on smartphones across all age groups, has heightened interest in understanding the potential health implications of prolonged digital media exposure within the Saudi population. Despite increasing research in this area, nationally relevant evidence examining the combined influence of smartphone use, social media behaviors, and lifestyle factors on mental well-being remains limited.

Therefore, the present study aimed to evaluate the relationship between smartphone use, social media engagement, and mental well-being among adults in Saudi Arabia using a nationwide cross-sectional survey. Specifically, we sought to examine whether greater smartphone and social media use were associated with poorer mental well-being, to identify behavioral factors independently associated with reduced psychological well-being, and to explore the potential protective influence of lifestyle factors such as physical activity and adequate sleep. We hypothesized that prolonged smartphone use, greater social media engagement, and problematic digital behaviors would be associated with lower mental well-being, whereas healthier lifestyle behaviors would be associated with more favorable psychological outcomes.

## Materials and methods

Study design and setting

This cross-sectional survey was conducted in the Kingdom of Saudi Arabia between January 2026 and March 2026 to examine the relationship between smartphone use, social media use, and mental well-being among adults.

Participants

Eligible participants were adults aged 18 years or older residing in Saudi Arabia who were able to read either Arabic or English and owned a smartphone. Individuals younger than 18 years, those who did not own a smartphone, and surveys with incomplete responses were excluded from the analysis.

Participants were recruited using an anonymous online questionnaire distributed through commonly used social media platforms, including WhatsApp, X (formerly Twitter), Instagram, Snapchat, and Telegram. To minimize duplicate responses, each participant was permitted to complete the survey only once using the survey platform's response restriction settings.

Survey instrument

The questionnaire was developed following a review of the published literature on smartphone use, social media engagement, and mental well-being. The first section collected demographic information, including age, sex, nationality, marital status, educational attainment, employment status, household income, smoking status, sleep duration, and physical activity (see Appendices).

The second section assessed smartphone use, including years of smartphone ownership, average daily smartphone use, frequency of checking the smartphone, primary purpose of use, smartphone use before sleep and after waking, and previous attempts to reduce smartphone use.

The third section evaluated social media use, including commonly used platforms, average daily time spent on social media, number of social media platforms used, primary purpose of social media use, posting frequency, social comparison behavior, experiences of cyberbullying, and perceived interference with work or academic performance.

The fourth section assessed problematic smartphone and social media behaviors using 10-point Likert-scale items evaluating behaviors such as compulsive checking, prolonged use beyond intended duration, fear of missing notifications, use for emotional regulation, and perceived negative effects on productivity and interpersonal relationships. Responses were recorded on a five-point Likert scale ranging from Never (1) to Always (5), with higher scores indicating greater problematic smartphone behavior.

Mental well-being was assessed in the fifth section using the WHO-5 Well-Being Index, a validated five-item instrument measuring positive psychological well-being over the preceding two weeks [17]. Each item was scored from 0 to 5, yielding a raw score ranging from 0 to 25, which was multiplied by four to generate a standardized score ranging from 0 to 100. Consistent with established recommendations, a WHO-5 score of <50 was considered indicative of poor mental well-being.

Data collection

The questionnaire was administered electronically using a secure online survey platform. Participation was voluntary and anonymous, and no personally identifiable information was collected. All survey items were mandatory to minimize missing data. Responses were automatically recorded and exported into a password-protected database accessible only to the study investigators.

Study variables

The primary outcome was mental well-being, measured using the WHO-5 score both as a continuous variable and as a binary outcome (poor mental well-being, defined as WHO-5 score <50). The primary exposure variables included average daily smartphone use, average daily social media use, smartphone use before bedtime, frequency of smartphone checking, social comparison on social media, and the composite problematic smartphone behavior score. Potential confounding variables included age, sex, marital status, educational level, employment status, smoking status, sleep duration, and physical activity.

Statistical analysis

Continuous variables were summarized as means and standard deviations or medians with interquartile ranges, depending on data distribution. Categorical variables were presented as frequencies and percentages. Differences in mental well-being across categories of smartphone use were evaluated using one-way analysis of variance (ANOVA) for continuous WHO-5 scores and the chi-square test for categorical outcomes. Linear trends across increasing categories of smartphone use were assessed where appropriate. Pearson correlation coefficients were calculated to evaluate associations between continuous measures of smartphone use, social media use, lifestyle factors, and WHO-5 scores. Multivariable logistic regression analysis was performed to identify independent factors associated with poor mental well-being. Variables considered clinically relevant or demonstrating statistical significance in univariable analyses were entered into the multivariable model. Results are reported as odds ratios (ORs) with 95% confidence intervals (CIs). Multicollinearity was assessed before model construction using variance inflation factors. All statistical tests were two-sided, and a P value of <0.05 was considered to indicate statistical significance. Statistical analyses were performed using IBM SPSS Statistics for Windows, Version 25 (Released 2017; IBM Corp., Armonk, New York, United States).

## Results

Participant characteristics

A total of 1,248 participants completed the survey and were included in the final analysis. The mean (±SD) age was 30.8±9.4 years, with the largest proportion of participants aged 25-34 years (35.3%), followed by 18-24 years (31.4%). Women comprised 691 (55.4%) of the study population, and 1,176 (94.2%) were Saudi nationals. Approximately three-quarters, 928 (74.4%), had attained at least a bachelor's degree, while 648 (51.9%) were employed and 404 (32.4%) were students. More than half of participants, 676 (54.2%), reported sleeping fewer than seven hours per night, whereas 493 (39.5%) engaged in regular physical activity at least three days per week. Current smoking was reported by 212 (17.0%) respondents (Table 1).

**Table 1:** Baseline demographic and lifestyle characteristics of the study participants Data are presented as numbers (percentages) unless otherwise indicated. Continuous variables are presented as mean ± standard deviation (SD). Percentages were calculated using the total study population (N=1,248) as the denominator. * Unmarried includes participants who were single, divorced, separated, or widowed.

Characteristic	Category	Overall (N = 1,248)
Age (year)	18-24	392 (31.4)
	25-34	441 (35.3)
	35-44	223 (17.9)
	45-54	132 (10.6)
	≥55	60 (4.8)
	Mean age (year)	30.8±9.4
Sex	Female	691 (55.4)
	Male	557 (44.6)
Nationality	Saudi	1,176 (94.2)
	Non-Saudi	72 (5.8)
Marital status	Married	476 (38.1)
	Unmarried*	772 (61.9)
Educational attainment	Less than a bachelor's degree	320 (25.6)
	Bachelor's degree or higher	928 (74.4)
Employment status	Employed	648 (51.9)
	Student	404 (32.4)
	Unemployed/Other	196 (15.7)
Smoking status	Current smoker	212 (17.0)
Sleep duration	<7 hr/night	676 (54.2)
Physical activity	≥3 days/week	493 (39.5)

Smartphone and social media use

Smartphone use was extensive across the study population. The median daily smartphone use was 5.8 hours (interquartile range, 3.9-8.2). Nearly half of participants, 543 (43.6%), reported using their smartphones for more than six hours daily. Daily social media engagement was similarly high, with 447 (35.8%) reporting at least five hours of social media use each day. Nearly three-quarters of respondents, 918 (73.6%), reported using their smartphone often or always within 30 minutes before bedtime, and 869 (69.6%) checked their smartphone within five minutes of waking. Approximately one-third, 404 (32.4%), frequently compared themselves with others on social media, while 389 (31.2%) reported that smartphone use interfered with work or academic performance. Additionally, 543 (43.5%) regularly used four or more social media platforms (Table 2).

**Table 2:** Patterns of smartphone and social media use among the study participants Data are presented as numbers (percentages) unless otherwise indicated. Daily smartphone use is summarized as the median (interquartile range (IQR)) because of its non-normal distribution. Responses regarding smartphone use before sleep, smartphone checking after waking, social comparison, interference with work or study, and the number of social media platforms used represent participants reporting the specified behavior.

Variable		n (%)
Daily smartphone use	<2 hr	106 (8.5)
	2-4 hr	262 (21.0)
	4-6 hr	337 (27.0)
	6-8 hr	289 (23.2)
	>8 hr	254 (20.4)
	Median smartphone use (IQR), hr/day	5.8 (3.9-8.2)
Daily social media use	<1 hr	123 (9.9)
	1-2 hr	275 (22.0)
	3-4 hr	403 (32.3)
	≥5 hr	447 (35.8)
	Smartphone use within 30 min before sleep (often/always)	918 (73.6)
	Smartphone checked within 5 min after waking	869 (69.6)
	Frequently compares self with others online	404 (32.4)
	Reports smartphone interferes with work/study	389 (31.2)
	Uses ≥4 social media platforms	543 (43.5)

Mental well-being according to smartphone use

Mental well-being demonstrated a clear inverse association with increasing daily smartphone use. Mean WHO-5 well-being scores progressively declined from 72.4±15.3 among participants using smartphones for less than two hours per day to 50.8±19.6 among those reporting more than eight hours of daily use.

Similarly, the prevalence of poor mental well-being (WHO-5 score <50) increased in a graded fashion across smartphone use categories, rising from 13 of 106 participants (12.3%) among individuals with less than two hours of daily smartphone use to 125 of 254 participants (49.2%) among those reporting more than eight hours per day. This dose-response relationship was statistically significant (P<0.001) (Table 3).

**Table 3:** Association between daily smartphone use and mental well-being ^†^ Poor mental well-being was defined as a World Health Organization Five Well-Being Index (WHO-5) score <50. WHO-5 scores are presented as mean ± standard deviation (SD). The percentages for poor mental well-being were calculated using the total number of participants within each smartphone-use category as the denominator: 106, 262, 337, 289, and 254, respectively. Differences in mean WHO-5 scores were assessed using one-way analysis of variance (ANOVA), and differences in the prevalence of poor mental well-being were assessed using the chi-square test. One-way ANOVA: F(4, 1243)=46.8, P<0.001; Chi-square test: χ^2^(4)=74.3, P<0.001

Daily smartphone use	WHO-5 score (mean±SD)	Poor mental well-being^†^, n/N (%)	P-value
<2 hr	72.4±15.3	13/106 (12.3)	<0.001
2-4 hr	68.9±16.1	47/262 (17.9)	
4-6 hr	63.5±17.4	89/337 (26.4)	
6-8 hr	57.3±18.8	109/289 (37.7)	
>8 hr	50.8±19.6	125/254 (49.2)	

Factors associated with poor mental well-being

In multivariable logistic regression analysis, adjusting for demographic characteristics and lifestyle factors, prolonged smartphone use remained independently associated with poor mental well-being. Participants reporting more than six hours of smartphone use daily had more than twice the odds of poor mental well-being compared with those reporting lower use (adjusted OR, 2.41; 95% CI, 1.77-3.28; P<0.001).

Likewise, spending at least five hours per day on social media was independently associated with poor mental well-being (adjusted OR, 2.05; 95% CI, 1.48-2.84; P<0.001). Frequent social comparison on social media demonstrated one of the strongest independent associations (adjusted OR, 2.36; 95% CI, 1.73-3.20; P<0.001). Smartphone use before bedtime also remained a significant predictor (adjusted OR, 1.69; 95% CI, 1.18-2.42; P=0.004).

Conversely, regular physical activity was independently associated with lower odds of poor mental well-being (adjusted OR, 0.71; 95% CI, 0.53-0.95; P=0.02). Female sex was not independently associated with poor mental well-being after adjustment (adjusted OR, 1.18; 95% CI, 0.90-1.54; P=0.23) (Table 4).

**Table 4:** Multivariable logistic regression analysis of factors associated with poor mental well-being Odds ratios (ORs) with 95% confidence intervals (CIs) are presented from univariable and multivariable logistic regression analyses. Poor mental well-being was defined as a WHO-5 score <50. The adjusted model included age, sex, educational attainment, employment status, smoking status, sleep duration, physical activity, daily smartphone use, and daily social media use. Wald χ^2^ statistics and corresponding two-sided P-values are reported for the adjusted model. WHO-5: World Health Organization Five Well-Being Index

Variable	Unadjusted OR (95% CI)	Adjusted OR (95% CI)	Wald χ^2^	P-value
Smartphone use >6 hr/day	3.21 (2.45-4.21)	2.41 (1.77-3.28)	29.4	<0.001
Social media use ≥5 hr/day	2.83 (2.14-3.74)	2.05 (1.48-2.84)	18.9	<0.001
Smartphone before sleep	2.16 (1.56-2.98)	1.69 (1.18-2.42)	8.2	0.004
Social comparison online	2.95 (2.24-3.88)	2.36 (1.73-3.20)	24.6	<0.001
Female sex	1.29 (1.01-1.66)	1.18 (0.90-1.54)	1.44	0.23
Sleep <7 hr	2.41 (1.86-3.13)	1.92 (1.42-2.58)	17.5	<0.001
Regular physical activity	0.63 (0.48-0.82)	0.71 (0.53-0.95)	5.4	0.020

Correlation between smartphone use and mental well-being

Correlation analyses further supported these findings. Greater problematic smartphone behavior demonstrated the strongest inverse correlation with WHO-5 well-being scores (r=-0.56, P<0.001). Significant negative correlations were also observed for daily smartphone use (r=-0.42), social comparison on social media (r=-0.44), smartphone use before bedtime (r=-0.37), smartphone checking frequency (r=-0.35), daily social media use (r=-0.39), and the number of social media platforms used (r=-0.18) (all P<0.001).

In contrast, longer sleep duration (r=0.29, P<0.001) and greater physical activity (r=0.22, P<0.001) were positively correlated with mental well-being (Table 5). Collectively, these findings demonstrate a consistent pattern in which greater smartphone and social media engagement - particularly problematic use and upward social comparison - is associated with poorer psychological well-being, whereas healthier lifestyle behaviors appear to exert a protective association.

**Table 5:** Correlation between smartphone-related behaviors and WHO-5 mental well-being scores Pearson correlation coefficients (r) were used to evaluate the relationship between smartphone-related behaviors and WHO-5 well-being scores. Positive correlation coefficients indicate an association with higher mental well-being scores, whereas negative coefficients indicate an association with lower mental well-being scores. Corresponding t-statistics and two-sided P-values are reported for each correlation analysis (degrees of freedom=1,246). WHO-5: World Health Organization Five Well-Being Index

Variable	Pearson r	t statistic	P-value
Smartphone hours/day	-0.42	-16.7	<0.001
Social media hours/day	-0.39	-15.2	<0.001
Number of social media platforms	-0.18	-6.5	<0.001
Smartphone checking frequency	-0.35	-13.4	<0.001
Smartphone use before sleep	-0.37	-14.2	<0.001
Social comparison	-0.44	-17.7	<0.001
Problematic smartphone behavior score	-0.56	-24.3	<0.001
Sleep duration	0.29	10.8	<0.001
Physical activity frequency	0.22	8.0	<0.001

## Discussion

In this cross-sectional survey of 1,248 adults in Saudi Arabia, we found a consistent inverse relationship between smartphone and social media use and mental well-being. Greater daily smartphone use, prolonged social media engagement, frequent social comparison, and bedtime smartphone use were all independently associated with poorer psychological well-being, even after adjustment for demographic and lifestyle factors. In contrast, regular physical activity was associated with better mental well-being. Furthermore, the observed dose-response relationship, whereby progressively greater smartphone use was accompanied by progressively lower WHO-5 well-being scores, strengthens the evidence for a graded association between digital media exposure and psychological health [17].

The prevalence of prolonged smartphone use observed in our study reflects the increasing integration of mobile technology into daily life. Nearly half of participants reported more than six hours of smartphone use each day, while almost three-quarters used their smartphones immediately before sleep. These findings are consistent with global trends demonstrating that smartphones have evolved from communication devices into platforms for work, education, entertainment, and social interaction [3-9]. In Saudi Arabia, where smartphone ownership and internet penetration are among the highest worldwide, extended daily exposure to digital media has become commonplace, particularly among younger adults. Although digital connectivity offers substantial educational and social benefits, excessive use may increase vulnerability to adverse psychological outcomes.

One of the principal findings of this study was the clear dose-response relationship between smartphone use and mental well-being. Participants reporting the highest levels of smartphone use exhibited substantially lower WHO-5 scores and nearly fourfold higher unadjusted odds of poor mental well-being compared with those reporting limited daily use. Importantly, these associations persisted after adjustment for several potential confounders, suggesting that prolonged smartphone use may contribute independently to diminished psychological well-being. Although causality cannot be inferred from a cross-sectional design, the consistency of the observed associations supports previous evidence linking excessive screen time with depressive symptoms, anxiety, emotional distress, and reduced life satisfaction [4-16].

Social media use demonstrated a similarly robust association with mental well-being. Participants spending at least five hours daily on social media had significantly higher odds of poor well-being after multivariable adjustment. While social media facilitates communication, access to information, and social connectedness, prolonged engagement may expose individuals to information overload, negative online interactions, unrealistic social comparisons, and continuous social evaluation. These mechanisms have been proposed as important contributors to emotional distress and reduced psychological well-being [2-8].

Among all behavioral variables examined, frequent social comparison demonstrated one of the strongest independent associations with poor mental well-being. This finding is consistent with social comparison theory, which proposes that repeated comparison with idealized representations of others may negatively influence self-esteem, body image, and overall psychological health. Modern social media platforms frequently promote highly curated content that may encourage upward social comparison and unrealistic expectations. The moderate inverse correlation observed between social comparison and WHO-5 scores in our study further supports the importance of this behavioral mechanism beyond simple duration of smartphone use [7-17].

Smartphone use immediately before sleep also emerged as an independent predictor of poor mental well-being. Several biological and behavioral mechanisms may explain this relationship [3,5,7]. Exposure to blue-wavelength light during the evening suppresses melatonin secretion and delays circadian rhythms, while emotionally stimulating digital content may increase cognitive arousal and prolong sleep latency [1,3,9]. Consistent with these mechanisms, more than half of our participants reported sleeping fewer than seven hours per night, and shorter sleep duration independently predicted poorer mental well-being. These findings underscore the interconnected relationship among smartphone use, sleep health, and psychological functioning.

Conversely, regular physical activity demonstrated a protective association with mental well-being. Participants engaging in exercise at least three days per week had significantly lower odds of poor psychological well-being after adjustment. This observation aligns with a large body of evidence demonstrating the beneficial effects of physical activity on mood regulation, stress reduction, sleep quality, and overall psychological resilience. The positive correlation between physical activity and WHO-5 scores in our cohort further reinforces the importance of healthy lifestyle behaviors as potential targets for preventive interventions.

Interestingly, female sex was associated with poorer mental well-being in unadjusted analyses but was no longer significant after multivariable adjustment. This suggests that the observed sex differences may be explained by differences in behavioral or lifestyle factors rather than sex itself. Such findings highlight the importance of considering modifiable behavioral characteristics, including digital media habits and sleep behaviors, when evaluating determinants of mental well-being [2,5,8].

The strengths of this study include the relatively large sample size, inclusion of participants from diverse demographic backgrounds, comprehensive assessment of smartphone and social media behaviors, and use of the validated WHO-5 Well-Being Index to assess psychological well-being. Additionally, the evaluation of multiple digital behaviors - including overall smartphone use, social media engagement, bedtime use, social comparison, and problematic smartphone behaviors - allowed a more comprehensive assessment of their independent relationships with mental well-being than studies focusing solely on screen time.

Several limitations should be acknowledged. First, the cross-sectional design precludes establishing temporal or causal relationships between smartphone use and mental well-being. It remains possible that individuals experiencing poorer psychological well-being are more likely to engage in excessive smartphone or social media use. Second, smartphone use and lifestyle variables were self-reported and therefore subject to recall bias and social desirability bias. Third, participants were recruited using an online convenience sampling strategy, which may limit generalizability and introduce selection bias toward individuals with greater digital engagement. Fourth, although several important confounders were adjusted for, residual confounding from unmeasured variables - including personality traits, socioeconomic factors, pre-existing psychiatric conditions, or life stressors - cannot be excluded. Finally, the WHO-5 is a screening instrument that measures positive psychological well-being rather than providing a clinical diagnosis of depression or anxiety.

The findings of this study have important public health implications. Given the widespread use of smartphones and social media in Saudi Arabia, promoting healthier digital habits may represent a practical strategy for improving population mental well-being. Public health initiatives and primary care counseling could emphasize limiting prolonged recreational smartphone use, reducing bedtime screen exposure, encouraging mindful social media engagement, and promoting regular physical activity and healthy sleep practices. Such interventions may be particularly relevant for younger adults, who represent the most intensive users of digital technologies.

## Conclusions

In this cross-sectional study of adults in Saudi Arabia, greater smartphone and social media use were independently associated with poorer mental well-being, with evidence of a clear dose-response relationship between increasing daily smartphone use and declining WHO-5 well-being scores. Behavioral factors, including prolonged social media engagement, frequent social comparison, and smartphone use before bedtime, were significant correlates of reduced psychological well-being, whereas regular physical activity was associated with more favorable mental health outcomes. Although the cross-sectional design precludes causal inference, these findings underscore the importance of promoting healthy digital habits as part of broader mental health and preventive care strategies. Future longitudinal studies incorporating objective measures of smartphone use are needed to clarify the temporal relationship between digital media exposure and psychological well-being and to determine whether interventions targeting modifiable digital behaviors can improve mental health outcomes.

## Appendices

**Table 6:** Survey questionnaire domains and variables used to assess smartphone use, social media behaviors, lifestyle factors, and mental well-being Data are presented as questionnaire domains, variables, and representative response options. Demographic characteristics included age, sex, nationality, marital status, educational attainment, employment status, household income, smoking status, sleep duration, and physical activity. Smartphone and social media behaviors assessed patterns of use, checking frequency, bedtime use, social comparison, and interference with work or study. Problematic smartphone behavior was evaluated using 10 Likert-scale items scored from 1 (Never) to 5 (Always), with higher scores indicating greater problematic use. Mental well-being was assessed using the World Health Organization Five Well-Being Index (WHO-5), comprising five items scored from 0 to 5 and converted to a standardized score ranging from 0 to 100 by multiplying the raw score by 4. Poor mental well-being was defined as a WHO-5 score <50.

Section	Variable	Example response options
Demographics	Age	18-24, 25-34, 35-44, 45-54, ≥55 years
	Sex	Male, Female
	Nationality	Saudi, Non-Saudi
	Marital status	Single, Married, Divorced, Widowed
	Education	Less than bachelor's, Bachelor's, Postgraduate
	Employment	Employed, Student, Unemployed, Other
	Household income	Income categories
	Smoking status	Current smoker, Former smoker, Never smoker
	Sleep duration	<5, 5-6, 7-8, >8 hours/night
	Physical activity	Days/week of exercise (0-7)
Smartphone Use	Years of smartphone ownership	Numeric or categories
	Average smartphone use/day	<2, 2-4, 4-6, 6-8, >8 hours
	Frequency of checking phone	Never, Rarely, Sometimes, Often, Always
	First phone check after waking	<5 min, 5-15 min, >15 min
	Smartphone use before bedtime	Never, Rarely, Sometimes, Often, Always
	Main purpose of smartphone use	Communication, Social media, Work/Study, Entertainment, Gaming, Other
	Attempted to reduce smartphone use	Yes/No
Social Media Use	Daily social media use	<1, 1-2, 3-4, ≥5 hours
	Platforms used	WhatsApp, X, Instagram, Snapchat, TikTok, Telegram, Facebook, Others
	Number of platforms used	1, 2-3, ≥4
	Primary purpose	Communication, News, Entertainment, Education, Business
	Posting frequency	Never to Daily
	Social comparison	Never, Rarely, Sometimes, Often, Always
	Experienced cyberbullying	Yes/No
	Smartphone/social media interferes with work or study	Never, Rarely, Sometimes, Often, Always
Problematic Smartphone Behavior (Likert scale: Never-Always)	Compulsive phone checking	1-5
	Use longer than intended	1-5
	Fear of missing notifications (FOMO)	1-5
	Use smartphone to improve mood	1-5
	Difficulty reducing use	1-5
	Distracted during work/study	1-5
	Phone interrupts conversations	1-5
	Feel anxious without phone	1-5
	Productivity reduced by phone use	1-5
	Relationships affected by phone use	1-5
Mental Well-Being (WHO-5)	Felt cheerful and in good spirits	0-5 (At no time → All of the time)
	Felt calm and relaxed	0-5
	Felt active and vigorous	0-5
	Woke up feeling fresh and rested	0-5
	Daily life filled with interesting things	0-5
